# HO-1 Induction by* Selaginella tamariscina* Extract Inhibits Inflammatory Response in Lipopolysaccharide-Stimulated RAW 264.7 Macrophages

**DOI:** 10.1155/2018/7816923

**Published:** 2018-11-18

**Authors:** An-Na Won, Sun Ah Kim, Jung Yun Ahn, Jae-Hyun Han, Chang-Hyun Kim, Ju-Hee Lee, Dong-Il Kim

**Affiliations:** ^1^Department of Korean Gynecology, College of Korean Medicine, Dongguk University, Goyang 10326, Republic of Korea; ^2^College of Korean Medicine, Dongguk University, Goyang 10326, Republic of Korea; ^3^College of Medicine, Dongguk University, Goyang 10326, Republic of Korea

## Abstract

*Selaginella Herba* is the dried, aerial part of* Selaginella tamariscina *(P.Beauv.) Spring and has been used to treat amenorrhea, abdominal pain, headaches, and hematuria in Korea. However, scientific evidence regarding the anti-inflammatory activity and action mechanism of* Selaginella tamariscina* is lacking. Thus, the present study was performed to investigate the anti-inflammatory and antioxidant activities of* Selaginella tamariscina* ethanol extract (STE) against lipopolysaccharide (LPS)-induced inflammatory responses and identify the molecular mechanism responsible. STE was prepared by heating in 70% ethanol and its quality was confirmed by HPLC. STE dose-dependently inhibited the productions of inflammatory mediators (NO and PGE_2_) and proinflammatory cytokines (IL-1*β* and IL-6) in LPS-stimulated RAW 264.7 cells. STE markedly suppressed the phosphorylations of MAPKs, I*κ*B-*α*, and NF-*κ*B and the nuclear translocation of NF-*κ*B induced by LPS stimulation. In addition, STE exhibited good free radical scavenging activity and prevented ROS generation by LPS. STE also upregulated the expression of Nrf2 and HO-1 and promoted the nuclear translocation of Nrf2. Taken together, STE was found to have anti-inflammatory and antioxidant effects on RAW 264.7 macrophages and the mechanism appeared to involve the MAPK, NF-*κ*B, and Nrf2/HO-1 signaling pathways. These results suggest that STE might be useful for preventing or treating inflammatory diseases and provide scientific evidence that supports the developments of herbal prescriptions or novel natural products.

## 1. Introduction

Inflammation is a manifestation of the response of immune systems to harmful stimuli, such as physical damage or damage caused by pathogens, and is characterized by increased blood flow to the damaged region, heat, redness, swelling, and pain [1]. Inflammation responses involve coordinated communications between different immune cells (macrophages, dendritic cells, T cells, B cells, and others) and blood vessels through a complex cascade of molecular signals [2]. Pattern recognition receptors, such as Toll-like receptors (TLRs) in macrophages, recognize and engage with pathogen-associated molecular patterns (PAMPs) like those of lipopolysaccharide (LPS; a major component of the outer membrane of gram-negative bacteria that binds with TLR4) and these interactions result in the activation of mitogen-activated protein kinase (MAPK) and nuclear factor-kappa B (NF-*κ*B) intracellular signaling pathways, which leads to the secretions of inflammatory mediators and proinflammatory cytokines [3]. If inflammation is not controlled, it may progress and become chronic, and systemic or chronic inflammation may be the root cause of many diseases including cancers, degenerative diseases, and obesity and can make people more susceptible to aging and disease [4, 5]. Accordingly, it is important that inflammation be treated promptly.

Nonsteroidal anti-inflammatory drugs (NSAIDs), such as naproxen, ibuprofen, and aspirin, are commonly prescribed for inflammation, but their long-term use may cause stomach ulcers, kidney damage, stroke, or heart attacks [6]. Corticosteroids are a class of steroid hormones that prevent a number of the mechanisms that underlie inflammation and are prescribed for a range of inflammation conditions. However, corticosteroids also have side effects, which include Cushing's syndrome, hypertension, hyperglycemia, osteoporosis, and connective tissue weakness [7]. Due to their efficacies and safety profiles, herbal medicines are being increasingly used as agents or supplements to control inflammation, and much research is being conducted to identify novel natural drugs.

*Selaginella Herba* (known in Korea as Kwon Baek or Boo Cheo Son) is the dried, aerial part of* Selaginella tamariscina *(P.Beauv.) Spring, a herbaceous evergreen and member of the Selaginellaceae family [8]. Because it is believed to promote blood circulation and restore menstrual flow, it has long been used to treat amenorrhea, abdominal pain, headaches, and asthma in Korea [9]. Belief in the therapeutic efficacy of* Selaginella tamariscina* is supported by clinical observations or Dongui Bogam, a representative text of Korean Medicine, but evidence-based data is lacking. Recent studies have demonstrated that* Selaginella tamariscina* has antiallergic, antihyperglycemic, and anticancer effects [9–12]. Previous studies have shown that the main bioactive components present in* Selaginella tamariscina* are bioflavonoids such as amentoflavone, hinokiflavone, isocryptomerine, sotetsuflavone, and sumaflavone [13, 14]. Furthermore, amentoflavone, sumaflavone, and lignan derivatives isolated from* Selaginella tamariscina* have been reported to inhibit the induction of inducible nitric oxide synthase (iNOS) and the production of nitric oxide (NO) [13, 15, 16]. However, no information is available in the literature about the anti-inflammatory or antioxidant activity of whole extract of* Selaginella tamariscina*. Therefore, in the present study, we examined the effects of* Selaginella tamariscina* extract (STE) on LPS-stimulated RAW 264.7 macrophages and sought to elucidate the mechanism responsible.

## 2. Materials and Methods

### 2.1. Chemicals and Reagents

Enzyme-linked immunosorbent assay (ELISA) kits for IL-1*β* and IL-6 were purchased from Ab Frontier (Seoul, Korea) and PGE_2_ was purchased from R&D Systems Inc. (Minneapolis, MN, USA). Primary antibodies, anti-COX-2, anti-HO-1, anti-iNOS, anti-I*κ*B-*α*, anti-p-I*κ*B-*α*, anti-NF-*κ*B (p65), anti-Nrf2, and secondary antibodies were purchased from Santa Cruz Biotechnology (Santa Cruz, CA, USA). Other primary antibodies were supplied by Cell Signaling Technology (Danvers, MA, USA). Dimethyl sulfoxide (DMSO) was purchased from Junsei Chemical Co. (Tokyo, Japan). LPS, amentoflavone, 3-(4,5-dimethylthiazol-2-yl)-2,5-diphenyl-tetrazolium bromide (MTT), Griess reagent, 2,2-diphenyl-2-picrylhydrazyl (DPPH), 4,6-diamidino-2-phenylindole (DAPI), and other reagents were purchased from Sigma-Aldrich (St. Louis, MO, USA).

### 2.2. Preparation of STE

Dried* Selaginella tamariscina *(P.Beauv.) Spring (*Selaginella Herba*, known in Korea as Kwon Baek or Boo Cheo Son) was purchased from Omniherb (Daegu, Korea) and authenticated by Professor Sun-Dong Park (Dongguk University, Gyeongju, Korea). Voucher specimens (DUMCKM2015-103) were deposited at the College of Korean Medicine, Dongguk University. Aerial parts (100 g) were extracted in 8-fold volumes of 70% ethanol (800 mL) at 80°C for 4 h. The extract was then filtered, concentrated using a rotary vacuum evaporator (EYELA, Japan), lyophilized using a freeze dryer (EYELA), and stored at 4°C. The yield of the lyophilized extract obtained was 16.5% (w/w) of dried* Selaginella tamariscina*.

### 2.3. High-Performance Liquid Chromatography (HPLC)

STE was analyzed using a Dionex Ultimate 3000 HPLC system (Thermo Fisher Scientific, Waltham, MA, USA) equipped with a binary solvent delivery pump, a vacuum degasser, and a diode array spectrophotometric detector for its amentoflavone (Sigma-Aldrich) content. Separation was performed using a VDSpher EC-C18 column (4.6 × 250 mm, 5 *μ*m, VDS optilab, Germany) at a column temperature of 30°C. The mobile phase consisted of mixtures of 0.3% trifluoroacetic acid (A) and acetonitrile (B), and gradient elution was performed using the following schedule: 0-1 min, 10% B; 1-25 min, 10-50% B; 25-35 min, 90% B; 35-40 min, and 90-10% B. The flow rate was maintained at 0.8 mL/min throughout and the injection volume was 10 *μ*L. The assays were monitored at 340 nm and chromatographic data were processed using Chromeleon 6.8 software.

### 2.4. Cell Culture and Cell Viability Assay

RAW 264.7 cells (a murine macrophage cell line) were obtained from the American Type Culture Collection (ATCC, Manassas, VA, USA) and cultured in Dulbecco's Modified Eagle's Medium (WELGENE, Gyeongsan, Korea), supplemented with 10% fetal bovine serum (WELGENE), 100 U/mL penicillin, and 100 *μ*g/mL streptomycin (Gibco, Grand Island, NY, USA). The cells were maintained at 37°C in a humidified 95% air and 5% CO_2_ incubator.

Cell viabilities were evaluated using an MTT assay. Briefly, RAW 264.7 cells were plated at a density of 1 × 10^4^ cells/well in a 96-well culture plate, incubated for 24 h, and treated with DMSO (control) or different concentrations of STE (10-300 *μ*g/mL) for 24 h. Viable cells were stained with MTT solution (final concentration 0.2 mg/mL) for 3 h, and the formazan crystals produced were dissolved by adding 100 *μ*L DMSO. Absorbances were measured at 540 nm using a microplate reader (Genios, Tecan, Austria).

### 2.5. Nitrite Assay

Nitrite concentrations in cultured macrophage supernatants were quantified using a Griess reagent test. Briefly, RAW 264.7 cells were plated at 4 × 10^5^ cells/well in 24-well culture plates, pretreated with different concentrations of STE (10-300 *μ*g/mL) for 1 h, and then stimulated with LPS (1 *μ*g/mL) for 24 h. Griess reagent [solution A: 1% sulfanilamide in 5% phosphoric acid, solution B: 0.1% N-(1-naphthyl)-ethylenediamine dihydrochloride in water; 1 : 1 (w/w)] was then mixed with equal volumes of cell supernatants and mixtures were allowed to stand at room temperature for 10 min. Absorbances were measured at 540 nm using a microplate reader.

### 2.6. ELISA

RAW 264.7 cells were seeded in 24-well culture plates at 4 × 10^5^ cells/well, pretreated with STE for 1 h, and then stimulated with LPS (1 *μ*g/mL) for 24 h. Inflammatory cytokine (IL-1*β*, IL-6, and PGE_2_) concentrations in culture supernatants were determined using ELISA kits following the manufacturer's protocol and were calculated from the standard curve for each cytokine.

### 2.7. DPPH Free Radical Scavenging Assay

The DPPH free radical scavenging activity of STE was assessed as described by Moreno, Isla, Sampietro, and Vattuone [17], with some modification. Briefly, different concentrations of STE (0, 10, 100, 200, or 300 *μ*g/mL) were added to 0.45 mL of 50 mM Tris-HCl buffer (pH 7.4) and then these mixtures were added to 1 mL of 0.1 mM DPPH in ethanol. Mixtures were then vortexed and incubated in the dark for 30 min at room temperature, and absorbances (abs.) were measured at 517 nm using a microplate reader. DPPH radical scavenging activity was calculated as follows.

(1) Scavenging  activity  %=control  abs.–sample  abs.control  abs.×100

### 2.8. Measurement of Intracellular Reactive Oxygen Species

Intracellular reactive oxygen species (ROS) levels were analyzed by DCFH-DA staining [18]. Briefly, RAW 264.7 cells were seeded on a 96-well black plate at 1 × 10^5^ cells/mL and incubated with LPS in the presence or absence of STE (10-300 *μ*g/mL). After removing medium, 10 *μ*M DCFH-DA in phosphate-buffered saline (PBS) was added to each well, and the plate was incubated for 30 min at 37°C. Fluorescence intensities were measured at 480 nm excitation/530 nm emission using a fluorescence microplate reader (Spectra Gemini, Molecular Devices).

### 2.9. Western Blotting

Total cellular proteins were extracted with radioimmunoprecipitation assay (RIPA) buffer (Thermo Scientific, Rockford, IL, USA) containing protease and phosphatase inhibitor cocktails (GenDEPOT, Barker, TX, USA). Cytoplasmic and nuclear lysates were separated using the NE-PER® nuclear and cytoplasmic extraction reagent kit (Thermos Scientific) by following the manufacturer's protocol. Briefly, equal amounts of protein (30-50 *μ*g) were separated by 10% sodium dodecyl sulfate-polyacrylamide gel electrophoresis and transferred to polyvinylidene difluoride membranes (EMD Millipore, Bedford, MA, USA), which were then blocked in PBST containing 5% skim milk for 1 h and incubated with primary antibodies (1:1000) overnight at 4°C. After three washes with PBST, horseradish peroxidase-conjugated secondary antibodies (1:5000) were added and membranes were incubated for 1 h at room temperature and rinsed. Detection was performed using an enhanced chemiluminescence prime solution (Amersham Bioscience, Buckinghamshire, UK). Bands were visualized using a Fusion Solo 2 M chemiluminescence imaging system (Vilber Lourmat, France).

### 2.10. Immunofluorescence Staining

RAW 264.7 cells were treated with STE (300 *μ*g/mL) in the absence or presence of LPS (1 *μ*g/mL) on 18 mm cover glasses in 12-well culture plates, rinsed 3 times with PBS, fixed with 100% methanol for 10 min, and rinsed again. Cells were then blocked with 1% bovine serum albumin, treated with NF-*κ*B p65 or Nrf2 antibody (1:200), and incubated overnight at 4°C. After extensive washing with PBS, cells were incubated with fluorescein isothiocyanate (FITC) conjugated secondary antibody (1:2000; Invitrogen, Carlsbad, CA, USA) for 1 h in room temperature and counterstained with DAPI (1:1000). After mounting cover glasses on glass slides using VECTASHIELD® HardSet™ Antifade Mounting Medium (Vector Laboratories, Burlingame, CA, USA), fluorescence images were captured using an inverted fluorescence microscope (ECLIPSE Ts2-FL; Nikon, Tokyo, Japan) equipped with a monochrome camera (DS-Qi2, Nikon).

### 2.11. Statistical Analysis

Results are expressed as means ± standard deviations (SD) of at least three independent experiments. Statistical significance was determined by one-way ANOVA with Tukey's multiple comparison test using GraphPad Prism software (GraphPad Software Inc., San Diego, CA, USA). Statistical significance was accepted for *p* values < 0.05.

## 3. Results

### 3.1. Effects of STE on Inflammatory Mediators in LPS-Stimulated RAW 264.7 Macrophages

**Figure 1:**
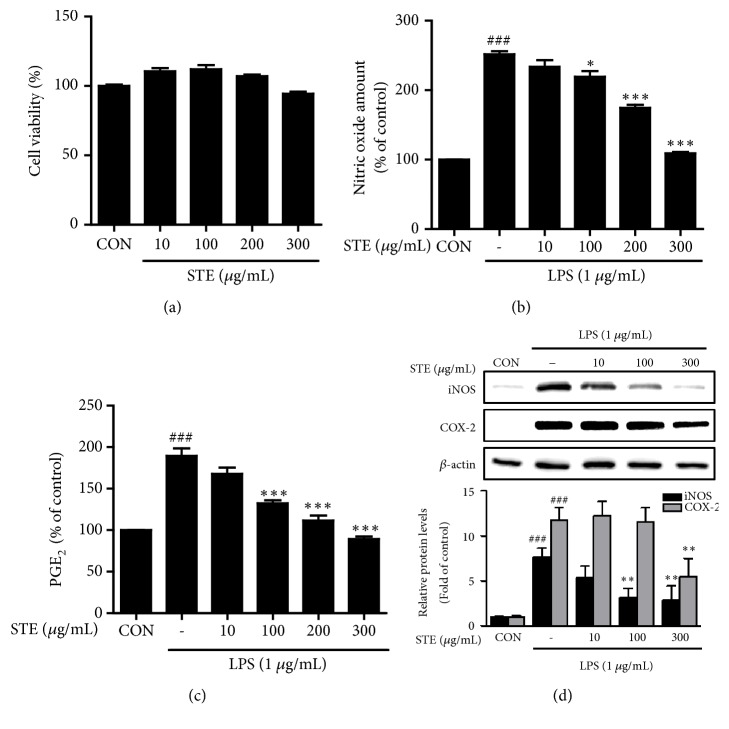
Effects of STE on the productions of inflammatory mediators in LPS-stimulated RAW 264.7 macrophages. (a) Effects of STE on the cell viability. (b) Effects of STE on LPS-induced NO production. (c) Effects of STE on LPS-induced PGE_2_ production. (d) Effects of STE on the induction of iNOS and COX-2 by LPS. RAW 264.7 macrophages were pretreated with different concentrations of STE (10, 100, or 300 *μ*g/mL) for 1 h and subsequently exposed to LPS (1 *μ*g/mL) for 24 h, and then protein expressions of iNOS and COX-2 were assessed by western blot. Statistical significance was determined versus vehicle-treated control group (^###^*P* < 0.001) or LPS-treated group (^*∗*^*P* < 0.05, ^*∗∗*^*P* < 0.01, and ^*∗∗∗*^*P* < 0.001).

An MTT assay was used to confirm STE had no toxic effect on RAW 264.7 macrophages. We found that STE at concentrations ranging from 10 to 300 *μ*g/mL had no effect on cell viability (Figure 1(a)). To investigate the anti-inflammatory effects of STE on LPS-stimulated RAW 264.7 macrophages, we measured amounts of NO and PGE_2_ secreted to medium. As shown in Figures 1(b) and 1(c), STE pretreatment at 100, 200, or 300 *μ*g/mL significantly reduced LPS-induced increase in NO and PGE_2_ secretions. In particular, pretreatment with 300 *μ*g/mL STE inhibited these LPS-induced increases to levels similar to those in nontreated control. Furthermore, western blotting for iNOS and COX-2 showed that LPS-induced iNOS increases were dose-dependently inhibited by STE. LPS-induced COX-2 increases were only inhibited by 300 *μ*g/mL STE (Figure 1(d)). These results indicated that STE effectively suppressed inflammatory mediators under LPS-induced inflammatory conditions in our* in vitro* model.

### 3.2. Effects of STE on the LPS-Induced Secretion of Proinflammatory Cytokines

**Figure 2:**
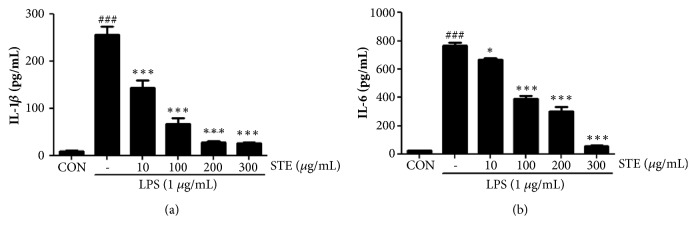
Effects of STE on the secretion of proinflammatory cytokines in LPS- stimulated RAW 264.7 macrophages. IL-1*β* (a) and IL-6 (b) levels were measured using ELISA kits. Statistical significance was determined versus vehicle-treated control group (^###^*P* < 0.001) or LPS-treated group (^*∗*^*P* < 0.05 and ^*∗∗∗*^*P* < 0.001).

To investigate the effect of STE on the LPS-induced secretion of proinflammatory cytokines, we checked IL-1*β* and IL-6 levels using ELISA kits. As shown in Figure 2, LPS stimulation significantly increased the levels of IL-1*β* and IL-6 to 255.8 ± 30.6 pg/mL (*P* < 0.01 versus control 9.9 ± 0.8 pg/mL) and 764.5 ± 37.2 pg/mL (*P* < 0.01 versus control 24.9 ± 2.6 pg/mL), respectively. However, STE pretreatment effectively inhibited the LPS-induced cytokines secretions (Figures 2(a) and 2(b)). In particular, at concentrations of 300 *μ*g/mL STE showed a strong suppressive effect on IL-1*β* and IL-6 secretion (26.9 ± 1.9 pg/mL and 57.3 ± 10.1 pg/mL, respectively) (Figure 2(b)).

### 3.3. Effects of STE on the Activation of the MAPK and NF-*κ*B Signaling Pathways

**Figure 3:**
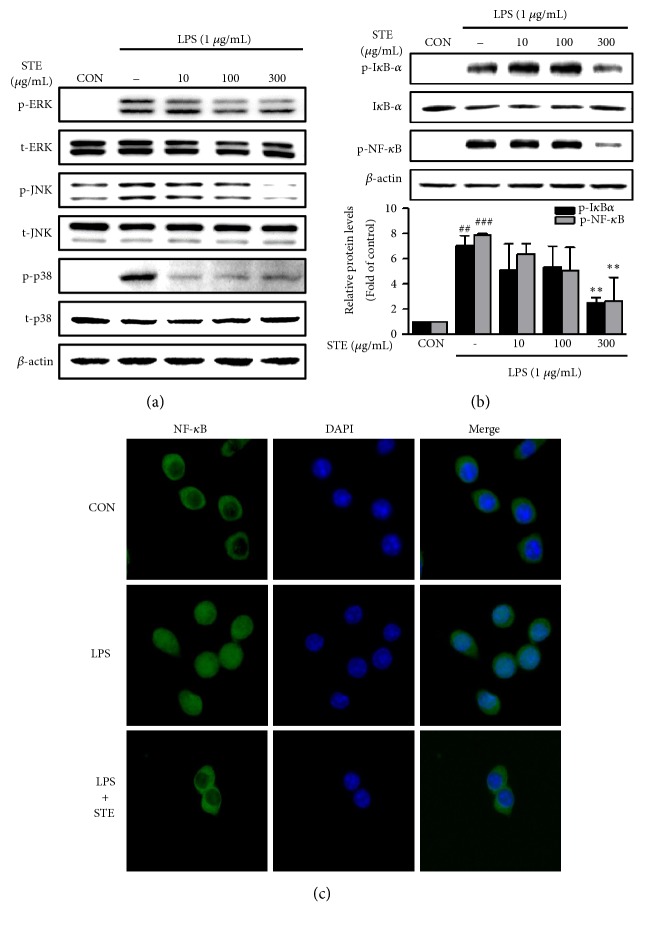
Effects of STE on the activation of MAPK and NF-*κ*B signaling pathway in LPS- stimulated RAW 264.7 macrophages. (a) Cells were pretreated with different concentrations of STE (10, 100, or 300 *μ*g/mL) for 12 h and then stimulated with LPS (1 *μ*g/mL) for 1 h. Cell lysates were western blotted to determine the protein levels of MAPKs (i.e., p-ERK, ERK, p-JNK, JNK, p-p38, and p38). (b) RAW 264.7 cells were pretreated with STE (300 *μ*g/mL) for 18 h and then stimulated with LPS (1 *μ*g/mL) for 1 h. Western blotting was performed using antibodies for p-I*κ*B-*α*, I*κ*B-*α*, and p-NF-*κ*B. Statistical significances were determined versus vehicle-treated control group (^##^*P* < 0.01 and ^###^*P* < 0.001), or LPS-treated group (^*∗∗*^*P* < 0.01). (c) The localization of NF-*κ*B p65 was visualized by fluorescence microscopy after immunofluorescence staining with NF-*κ*B p65 antibody (green) and DAPI to visualize nuclei (blue).

MAPKs (ERK1/2, JNK, and p38) and NF-*κ*B are important transducers of inflammation [19]. To understand the mechanisms responsible for the anti-inflammatory effects of STE, we investigated the effects of STE on the MAPK signaling pathway by western blotting. LPS stimulation of RAW 264.7 cells significantly induced the phosphorylations of ERK, JNK, and p38 MAPK, but STE pretreatment dose-dependently inhibited these phosphorylations (Figure 3(a)). In particular, at a concentration as low as 10 *μ*g/mL STE significantly inhibited p38 MAPK activation.

Next, we analyzed the expressions of I*κ*B-*α* and NF-*κ*B by western blotting to determine whether STE affects NF-*κ*B activation. LPS strongly induced the phosphorylated forms of I*κ*B-*α* and NF-*κ*B in RAW 264.7 cells, but pretreatment with STE at 300 *μ*g/mL effectively inhibited these phosphorylations (Figure 3(b)). Since the phosphorylation of NF-*κ*B p65 leads to its nuclear translocation and the subsequent transcriptions of target genes [20], we confirmed that STE inhibited the LPS-induced nuclear translocation of NF-*κ*B p65 by immunofluorescent staining (Figure 3(c)).

### 3.4. Effects of STE on Antioxidant Activity

**Figure 4:**
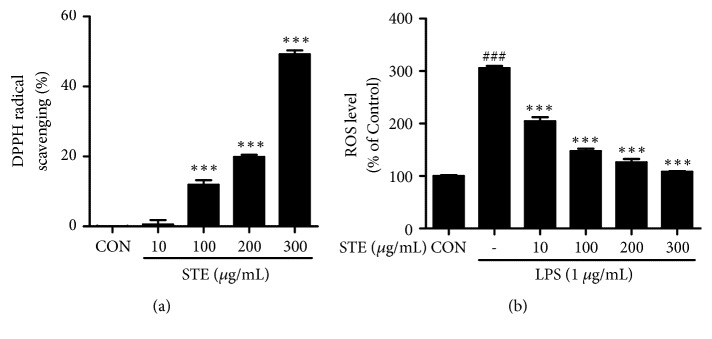
Antioxidant effects of STE in RAW 264.7 macrophages. (a) DPPH free radical scavenging activity. (Significant vs. vehicle-treated control, ^*∗∗∗*^*p* < 0.001). (b) Effects of STE on ROS generation. Cells were stimulated with LPS (1 *μ*g/mL) in the absence or presence of various concentrations of STE (10, 100, 200, or 300 *μ*g/mL) for 24 h. Intracellular ROS levels were determined by measuring DCF fluorescence intensities. Statistical significances were determined versus vehicle-treated control group (^###^*P* < 0.001), or LPS-treated group (^*∗∗∗*^*P* < 0.001).

Oxidative stress can cause various types of protein oxidation, which results in inflammation [21]. DPPH free radical scavenging assay showed that STE had a strong scavenging activity in a dose-dependent manner (Figure 4(a)). Furthermore, LPS stimulation increased ROS levels about 3-fold in RAW 264.7 cells and this increase was dose-dependently inhibited by STE pretreatment, and at 300 *μ*g/mL this LPS-induced increase was completely blocked (Figure 4(b)).

### 3.5. Effects of STE on Nrf2/HO-1 Pathway Activation

**Figure 5:**
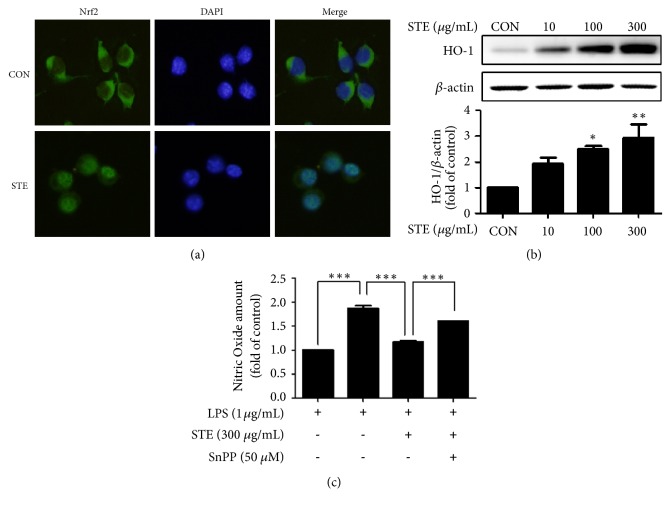
Induction of Nrf2/HO-1 signaling pathway by STE. (a) Immunofluorescence images of the nuclear translocation of Nrf2 induced by STE. RAW 264.7 cells were treated with STE (300 *μ*g/mL) for 3 h. (b) Induction of HO-1 by STE. RAW 264.7 cells were treated with various concentrations of STE (10, 100, or 300 *μ*g/mL) for 18 h, lysed, and western blotted. (Significant vs. vehicle-treated control, ^*∗*^*p* < 0.05 and ^*∗∗*^*p* < 0.01). (c) Blocking of the NO inhibitory effect of STE on LPS-induced NO production by SnPP. RAW 264.7 cells were pretreated with STE (300 *μ*g/mL) for 1 h in the presence or absence of SnPP (50 *μ*M, 30 min) and then stimulated with LPS (1 *μ*g/mL) for 18 h. (^*∗∗∗*^*p* < 0.001).

The induction of cytoprotective and antioxidant genes via Nrf2 activation is a major mechanism in the cellular defense against oxidative stress [22]. Therefore, we investigated whether free radical scavenging activity and inhibitory effect of ROS generation by STE treatment were correlated with induction of Nrf2 and its target gene. Immunofluorescence staining for Nrf2 showed that at 300 *μ*g/mL STE induced the nuclear translocation of Nrf2 (Figure 5(a)). Next, we examined whether STE affects the expression of HO-1, a downstream target gene of Nrf2. STE dose-dependently upregulated the protein expression of HO-1 and peak expression was observed for 300 *μ*g/mL STE (Figure 5(b)). A NO assay was performed by pretreating RAW 264.7 cells with SnPP (50 *μ*M; an inhibitor of HO) for 30 min before treating them with STE (300 *μ*g/mL) and then LPS, and results showed the inhibition of LPS-induced NO production by STE blocked by SnPP (Figure 5(c)). These observations suggest that the observed anti-inflammatory effect of STE was associated with activation of the Nrf2/HO-1 axis.

### 3.6. HPLC Analysis of STE

**Figure 6:**
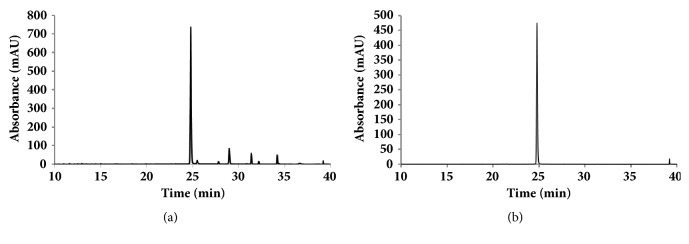
HPLC chromatogram of STE (a) and amentoflavone (b). HPLC chromatogram identified the STE peak with a retention time of 24.79 min as amentoflavone.

Amentoflavone content in STE was used as a HPLC quality control marker. Data from the HPLC analysis of STE were recorded in the form of chromatograms.  Figure 6 shows HPLC chromatograms of STE and amentoflavone as determined using a 340 nm detector and identified the STE peak with a retention time of 24.79 min as amentoflavone. The content of amentoflavone in STE was calculated from the calibration curve of the standard, and it was 37.3 mg/g.

## 4. Discussion

Acute inflammation is a symptom of a bacterial or viral infection, whereas chronic inflammation progresses slowly without obvious symptoms and can result in diseases, such as hypertension, diabetes, and cancer [23]. Chronic inflammation has many life styles related causes, which include eating habits, stress, and environmental pollution [24]. According to Korean medicine,* Selaginella Herba* (the dried aerial part of* Selaginella tamariscina *Spring) cleanses blood, stops bleeding, resolves phlegm, relieves the symptoms of asthma, promotes urination, and can be used to treat boils [8]. Furthermore, the effects of* Selaginella Herba* are believed to depend on the form used; that is, the raw herb is used to cleanse blood, relieve asthma symptoms, and treat amenorrhea, while the roasted form is used to stop bleeding and treat prolapsed rectum [8]. It has been frequently prescribed as a remedy in traditional Korean medicine for various diseases at a dose of 3–9 g [8]. As traditional Korean medicine incorporates several herbs simultaneously in prescriptions, studying the efficacy and mechanism of each herbal source is required and plays a major role in developing new prescriptions. Although the anti-inflammatory effects of several important bioactive components present in* Selaginella tamariscina* have been reported, scientific evidence regarding the efficacy and mechanism of action of whole extract of* Selaginella tamariscina* is lacking, and, thus, we conducted this study to investigate anti-inflammatory effects of STE using a LPS-stimulated RAW 264.7 cell model and to determine the nature of the mechanism involved, as part of a basic study to develop new herbal prescriptions or new natural products.

LPS is commonly used to model inflammation, and it induces macrophage activation via TLR4-mediated signal transduction, which results in the productions and secretions of inflammatory mediators (e.g., NO and PGE_2_) and proinflammatory cytokines (e.g., IL-1*β*, IL-6, and TNF-*α*) by activating the MAPK and NF-*κ*B intracellular signaling pathways [25]. NO is synthesized endogenously during the conversion of L-arginine to L-citrulline by one of the three isoforms of nitric oxide synthase (NOS) [26]. Excessive amounts of NO by iNOS in macrophages are cytotoxic and cause organ injury and inflammation, and iNOS inhibitors have been demonstrated to have anti-inflammatory properties [27]. PGE_2_ is generated from arachidonic acid due to the action of COX-2 induced by inflammatory stimuli, hormones, or growth factors and plays a key role in inflammatory response [28]. Thus, COX-2 is a target of NSAIDs. In the present study, STE was found to dose-dependently inhibit the productions of NO and PGE_2_ in LPS-stimulated RAW 264.7 cells and to suppress the expressions of iNOS and COX-2 (Figure 1). Furthermore, STE significantly prevented the LPS-induced secretions of IL-1*β* and IL-6 (Figure 2). Consistent with our results, Kuo et al. reported that crude extract of* Selaginella tamariscina* reduces the productions of proinflammatory cytokines, IL-1*β* and tumor necrosis factor-*α*, in human mesangial cells, which play an important role in inflammatory reactions in kidneys [29].

The MAPK and NF-*κ*B intracellular signaling pathways play key roles in the production of inflammatory mediators in macrophages [30]. In the present study, pretreatment with STE significantly and dose-dependently reduced the phosphorylations of ERK1/2, p38, and JNK, which suggested that STE inhibited the activation of MAPK signaling (Figure 3(a)). Activation of the NF-*κ*B pathway leads to phosphorylation and ubiquitin-dependent degradation of I*κ*B*α* (a NF-*κ*B inhibitor) and promotes the nuclear translocation of NF-*κ*B p65, which results in the productions of proinflammatory cytokines, chemokines, and other mediators of inflammation [31]. In the present study, pretreatment with STE reduced the nuclear translocation of NF-*κ*B p65 by inhibiting the phosphorylations of I*κ*B*α* and NF-*κ*B p65 (Figures 3(b) and 3(c)). STE blocks the NF-kB pathway only at 300 *μ*g/mL, whereas proinflammatory cytokines such as IL-1*β* and IL-6 are significantly inhibited at a concentration as low as 10 *μ*g/mL. This effect may be attributed to the fact that STE is more sensitive to the inhibition of p38 MAPK phosphorylation among activated MAPKs by LPS stimulation. Woo et al. reported that amentoflavone inhibits LPS-induced NO production by inhibiting NF-*κ*B activation in RAW 264.7 cells [15]. Interestingly, this paper reported that amentoflavone pretreatment did not affect the LPS-induced phosphorylations of ERK or JNK. Sumaflavone, which is also present in* Selaginella tamariscina*, has been reported to suppress iNOS-mediated NO production in LPS-treated macrophages, but this suppression was found to be associated with the blocking of AP-1 activation, and not NF-*κ*B activation [13]. Therefore, it appears that STE may have acted on multiple targets to inhibit inflammatory responses in LPS-stimulated RAW 264.7 cells, because it contains a variety of components.

Endogenous free radicals are generated by immune cell activation, inflammation, ischemia, infection, cancer, and aging [32]. Since, free radicals are highly unstable molecules that can react rapidly with various organic substrates, their excessive accumulation* in vivo* causes oxidative stress [33]. The human body can counteract oxidative stress using endogenous or exogenous antioxidants [34]. Antioxidants act as “free radical scavengers”, prevent and facilitate the repair damage caused by ROS, and thus enhance immune defenses and lower the risk of chronic disease [32]. The present study shows that STE has excellent DPPH free radical scavenging activity and that it dose-dependently inhibits LPS-induced ROS increases in RAW 264.7 cells (Figures 4(a) and 4(b)).

Nrf2 is a transcription factor and responsible for the regulation of cellular redox balance and protective antioxidant and phase II detoxification responses in mammals [35]. Under normal conditions, Nrf2 is sequestered as a silent form in cytoplasm by binding with Keap1, its repressor protein [36]. However, under stressful conditions, Nrf2 dissociates from Keap1 and translocates to the nucleus where it binds to conserved the antioxidant response element (ARE) sequences in the promoter regions of detoxifying genes (cytoprotective Nrf2-regulated genes, which include HO-1, GST, and NQO-1) [37]. Recently, it has been suggested that pharmacological manipulation of the Nrf2/HO-1 axis might have important implications in the context of relieving inflammation [38], and thus we considered that the anti-inflammatory effects of STE might associate with the Nrf2/HO-1 pathway. We found that STE treatment significantly and dose-dependently induced HO-1 expression (Figure 5(b)) and that blockade of HO-1 induction using SnPP suppressed inhibitory effect of STE on LPS-induced NO production in RAW 264.7 cells (Figure 5(c)). These observations suggest that the anti-inflammatory effect of STE is associated at least in part with activation of the Nrf2/HO-1 axis and is meaningful because there has not yet been a study on the induction of HO-1 in* Selaginella tamariscina* or its constituents. Several studies have shown that compounds from plants such as curcumin, isothiocyanates, and anthocyanins are not only good antioxidants but also potent anti-inflammatory agents acting via Nrf2 induction [39–42]. These compounds activate the Nrf2 signaling pathway principally in the form of electrophiles modifying the cysteine residues of Keap1 [42]. In addition, other ROS-independent pathways can regulate Nrf2, i.e., a wide variety of kinase signaling pathways such as protein kinase C (PKC), MAPKs, phosphatidylinositol 3-kinase (PI3K), and PKR-like endoplasmic reticulum kinase (PERK) [43]. Ho et al. suggested that Nrf2 activation in the absence of oxidative stress might be involved with ROS-independent ER stress pathway by upregulating the ER responsive genes gadd34, gadd45, gadd153, and ndr1 in tetrafluoroethylcysteine-induced cytotoxicity [43]. Thus, STE may elicit anti-inflammatory effects by activating Nrf2 or by mediating ROS-independent ER stress pathway and scavenging ROS under the LPS-induced inflammatory conditions.

## 5. Conclusion

This study shows that STE has antioxidant activity and reduces LPS-induced inflammatory responses in RAW 264.7 cells and suggests that this effect of STE is due to inhibitions of the activation of NF-*κ*B and MAPK and the promotion of Nrf2/HO-1 activation. These results highlight the potential utility of STE as a phytomedicine for preventing or curing inflammation, and it may be used as a foundation for developing new prescriptions in the future, playing a role in the evolution of “evidence-based medicine” in Korean medicine. To confirm these findings, animal studies are needed to investigate the toxicological and the efficacy of STE in different inflammatory diseases. The relevant human dose can be obtained by a detailed animal scale-up study in the future.

## Data Availability

The data used to support the findings of this study are available from the corresponding author upon request.
